# Micro-Shaping of Pure Aluminum in Long-Duration Wire Electrochemical Micromachining Using Bipolar Nanosecond Pulses

**DOI:** 10.3390/mi14051046

**Published:** 2023-05-13

**Authors:** Xiaolei Bi, Meng Jia, Lingchao Meng

**Affiliations:** 1 School of Mechanical Engineering, Henan Institute of Technology, Xinxiang 453003, China; 2 School of Mechanical and Electrical Engineering, Xinxiang University, Xinxiang 453003, China; 3 College of Civil Aviation, Northwestern Polytechnical University, Xi’an 710072, China; lcmeng@nwpu.edu.cn; 4 Yangtze River Delta Research Institute of NPU, Northwestern Polytechnical University, Taicang 215400, China

**Keywords:** wire electrochemical micromachining, machining accuracy, machining stability, pure aluminum, bipolar nanosecond pulses

## Abstract

With the increasing application of three-dimensional pure aluminum microstructures in micro-electromechanical systems (MEMS) and for fabricating terahertz components, high-quality micro-shaping of pure aluminum has gradually attracted attention. Recently, high-quality three-dimensional microstructures of pure aluminum with a short machining path have been obtained through wire electrochemical micromachining (WECMM), owing to its sub-micrometer-scale machining precision. However, machining accuracy and stability decrease owing to the adhesion of insoluble products on the surface of the wire electrode in long-duration WECMM, which limits the application of pure aluminum microstructures with a long machining path. In this study, the bipolar nanosecond pulses are used to improve the machining accuracy and stability in long-duration WECMM of pure aluminum. A negative voltage of −0.5 V was considered appropriate based on experimental results. Compared with the traditional WECMM using unipolar pulses, the machining accuracy of the machined micro-slit and the duration of stable machining were significantly improved in long-duration WECMM using bipolar nanosecond pulses.

## 1. Introduction

Pure aluminum possesses numerous special characteristics, such as low density, high electrical conductivity, good thermal properties, and excellent corrosion resistance. Therefore, it can be manufactured into special components, such as reflective micromirror arrays, micromotors, and micro-rotors for use in microelectromechanical systems (MEMS) [1]. Recently, pure aluminum microstructures have also been applied in manufacturing terahertz microcavity components. For example, high-working-frequency terahertz hollow-core rectangular metal cavities can be manufactured using a high-quality pure aluminum sacrificial mandrel and its selective chemical dissolution [2].

However, there are still some gaps in the existing micromachining technology for high-precision fabrication of three-dimensional pure aluminum microstructures. Mechanical micromachining will cause plastic deformation and surface consistency problems owing to the low hardness, low strength, high ductility, and high plasticity of pure aluminum [3]. Electrical discharge micromachining and laser micromachining will inevitably produce defects, such as the recast layer, residual thermal stresses, and the heat-affected zone [4,5]. Although electrochemical machining technology has realized the machining of various types of hydrophilic pure aluminum microstructures, these microstructures are usually machined on the surfaces of pure aluminum substrates [6,7].

Based on the principle of electrochemical anode dissolution, wire electrochemical micromachining (WECMM) uses a microscale wire as the tool cathode, controls the movement track of the wire electrode or metal workpiece through programmable software, and realizes the machining of microstructures such as microgrooves, micro-slits, and three-dimensional microstructures with complex shapes or high aspect ratios [8]. WECMM has the following advantages: a low machining temperature, no stress on the machining surface, no metamorphic layer, no tool loss, and is independent of the hardness of parts and materials [9,10]. Therefore, WECMM is an ideal technique to manufacture three-dimensional microstructures from pure aluminum. WECMM has been successful for fabricating high-quality three-dimensional microstructures from a wide range of materials, such as pure nickel [11], stainless steel [12], cobalt-based alloys [13], and zr-based amorphous alloys [14]. Recently, high-quality three-dimensional micromachining of pure aluminum has been achieved through WECMM, but this is possible only in short-duration WECMM [2]. With the increasing demand for long-track three-dimensional microstructures of pure aluminum, it is extremely important to improve the machining stability and machining accuracy in long-duration WECMM of pure aluminum.

In long-duration WECMM, machining stability and machining accuracy will be reduced, as observed during the WECMM of some metal materials, such as pure aluminum, stainless steel, and metallic glass [2,15,16]. During long-duration WECMM of long-track microstructures, the bubbles and insoluble electrolysis products discharged from the machining gap do not diffuse easily owing to the limited mass transfer in the current WECMM process. They gradually accumulate in the machining gap area, which will further complicate the mass transfer through the microscale gap and the desorption of bubbles on the surface of the wire electrode. At the same time, the accumulated insoluble products easily adhere to the wire electrode surface under the action of the electric field force. After machining for a particular period, frequent instantaneous short circuits will occur, and the machining accuracy will deteriorate. Researchers have proposed some methods to solve this problem. Bi et al. proposed an enhanced mass transfer method involving intermittent ultrasonic vibration, which realized the timely dispersion of bubbles and insoluble products in the machining process and improved the machining stability and machining accuracy [2]. Meng et al. proposed a WECMM process using bipolar pulses [15]. This process inhibited the adhesion of products to the surface of the wire electrode and improved the machining stability and accuracy, which was also confirmed by Gao et al. [16]. In addition, Xu et al. used hydrochloric acid as the electrolyte to reduce the production of insoluble electrolytic products [17].

In this study, bipolar pulses were used to reduce the deposition of insoluble products on the wire electrode surface in long-duration WECMM of pure aluminum. The machining principle was analyzed in detail. The effectiveness of using bipolar nanosecond pulses for improving the machining stability and accuracy in long-duration WECMM of pure aluminum was verified by systematic experiments.

## 2. Materials and Methods

### 2.1. Materials

In this study, a workpiece of pure aluminum (99.99%; Goodfellow Ltd., Huntingdon, UK) with a thickness of 90 μm was ultrasonically cleaned before the WECMM, a 20 μm-diameter tungsten wire (Goodfellow Ltd., Huntingdon, UK) was adopted as the cathode, and the electrolytes were prepared from analytical-grade NaNO_3_ and NaCl using deionized water. According to a previous study by Bi et al. [2], a mixed electrolyte of 0.0125 mol/L of NaNO_3_ and 0.00625 mol/L of NaCl was selected as an appropriate electrolyte for the WECMM of pure aluminum.

### 2.2. Methods

**Figure 1:**
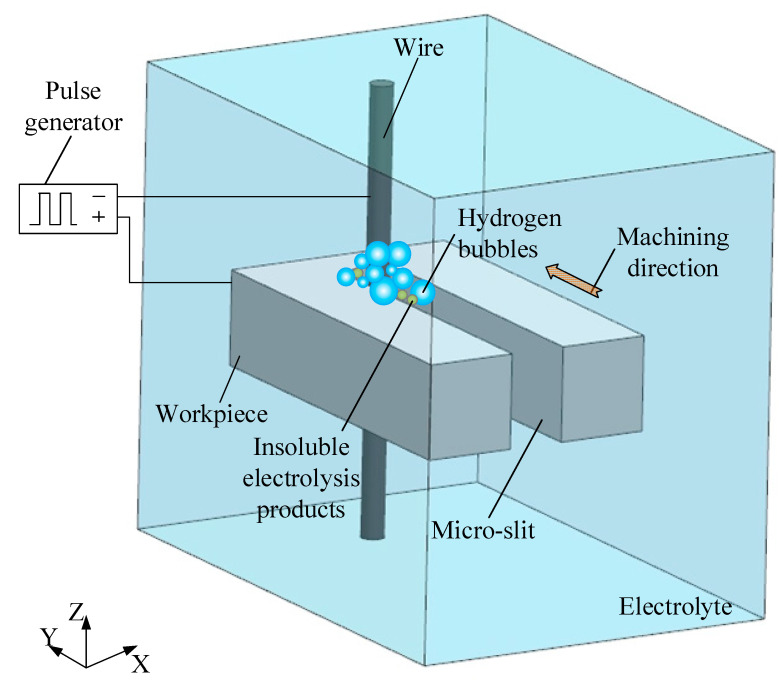
Schematic of traditional WECMM using unipolar pulses.

As shown in Figure 1, the traditional WECMM with unipolar pulses uses a nanosecond pulse generator as the power supply, a microscale metal wire as the tool cathode, and a metallic workpiece as the anode. The electrolyte used depends on the electrochemical dissolution characteristics of the machined metals. During machining, the metal workpiece near the wire cathode is dissolved and removed in a certain range, accompanied by the production of insoluble electrolysis products and the precipitation of hydrogen bubbles on the surface of the wire cathode. Through control using programmable software, the wire electrode or workpiece are continuously fed along a predetermined path, and the desired metal micro-structures are obtained.

**Figure 2:**
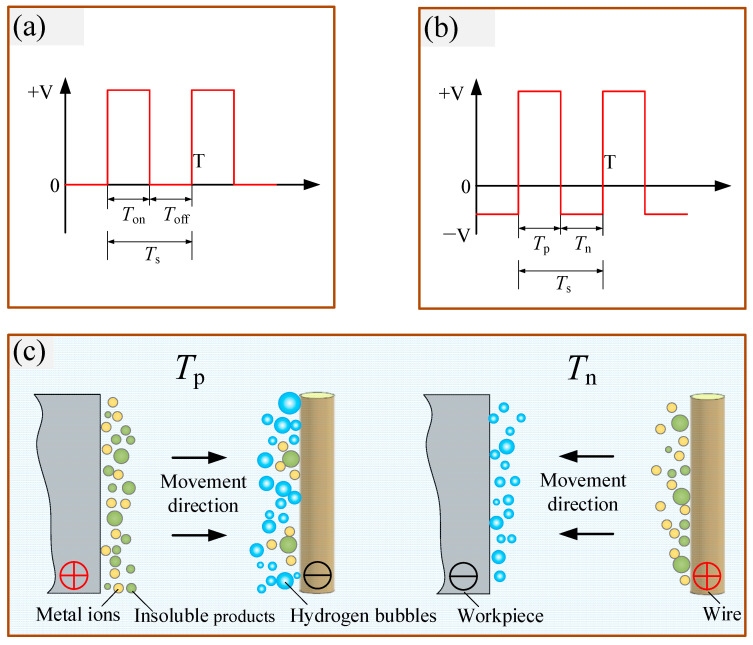
Comparison of pulse shapes between the unipolar pulse and bipolar pulse: (**a**) unipolar pulse, (**b**) bipolar pulse, and (**c**) principle of mass transfer during WECMM using bipolar pulses. *T*_on_ is the pulse-on time; *T*_off_ is the pulse-off time; *T*_p_ is the positive pulse-on time; *T*_n_ is the negative pulse-on time; *T*_s_ is the pulse period.

Figure 2a,b compare the shapes of the unipolar and bipolar pulses. In traditional WECMM using unipolar pulses, the electrochemical reaction occurs during the positive pulse-on time, as shown in Figure 2a. In traditional WECMM using bipolar pulses, as the electrode polarities of the workpiece and wire alternately change, the electrochemical reaction occurs not only in the positive pulse-on time but also in the negative pulse-on time, as shown in Figure 2b.

Figure 2c shows the principle of mass transfer during WECMM using bipolar pulses. During the positive pulse-on time, the metal ions and insoluble products generated by electrochemical dissolution of the workpiece anode move toward the surface of the wire electrode, owing to the electric field force. With continuous machining, the insoluble products will adhere to the surface of the wire electrode, leading to reduced mass transfer efficiency in the narrow micromachining gap. During the negative pulse-on time, the insoluble products on the surface of the wire electrode will be repulsed owing to the electric field force, which will inhibit the adhesion of insoluble products on the surface of the wire electrode. Under the action of the axial reciprocating vibration of the wire electrode, using a negative pulse can enable easy discharge of the insoluble products from the machining gap, thereby improving the mass transfer efficiency. Due to the improvement of mass transfer efficiency in the long-duration wire electrochemical micromachining using bipolar nanosecond pulses, more fresh electrolytes are brought into the machining area through the reciprocating vibration of the wire, the conductivity of the machining area will become more uniform, and the electrochemical dissolution of the anode material in the machining area will also become more uniform, so the machining stability and accuracy are improved. However, as the wire electrode is positive with respect to the workpiece during the negative pulse duration, the wire electrode itself may also be electrochemically dissolved if it is fabricated from a metallic material.

## 3. Experimental

**Figure 3:**
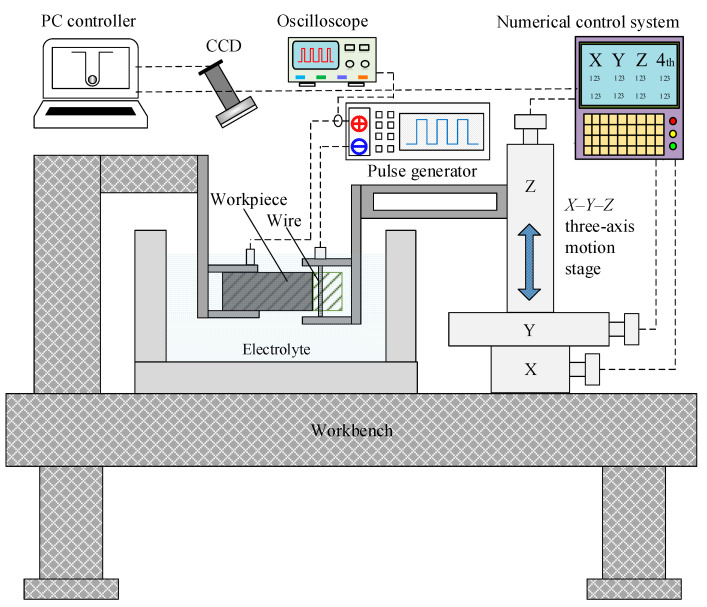
Schematic of the WECMM system.

The WECMM system for this study is shown in Figure 3. The experimental system consists of a PC controller, a three-axis motion stage, a digital relay, a pulse generator, an ultrasonic generator, an oscilloscope, an electrolyte tank, the tungsten wire, and the workpiece. Table 1 lists the experimental conditions selected based on the study by Bi et al. [2]. 

The topographies of the experimental samples were examined by scanning electron microscopy (SEM), and the elemental composition of the insoluble products was investigated by energy-dispersive X-ray spectroscopy (EDX). During machining, the behavior of the bubbles in the machining area was observed and recorded using a computer-controlled digital camera (CCD).

## 4. Results

### 4.1. Comparison between Short- and Long-Duration WECMM

**Figure 4:**
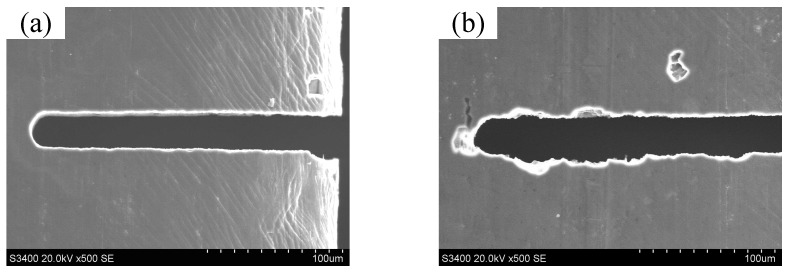
Comparison of the micro-slits obtained by WECMM using a traditional unipolar pulse: (**a**) short-duration WECMM spanning approximately 30 min and (**b**) long-duration WECMM spanning approximately 6.1 h.

**Figure 5:**
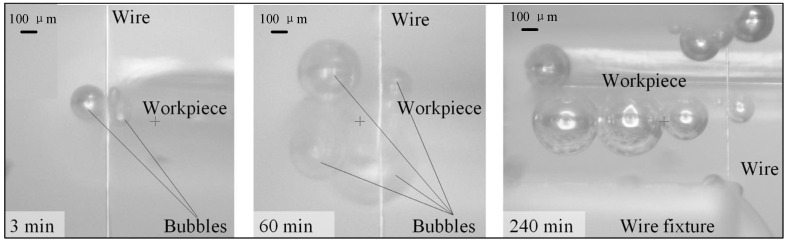
CCD images captured during long-duration WECMM using a traditional unipolar pulse.

**Figure 6:**
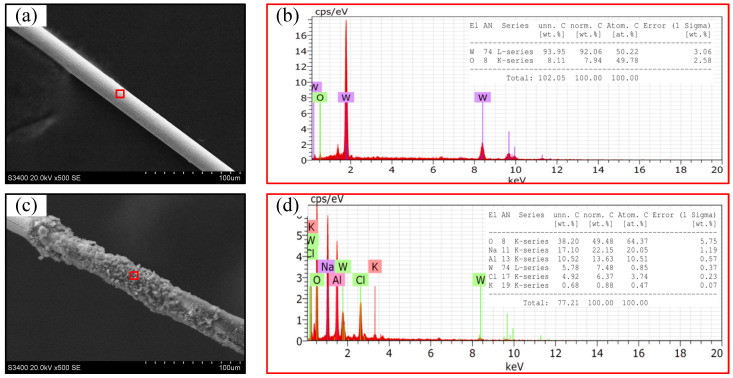
(**a**) SEM image of the wire before WECMM and (**b**) corresponding EDX results for the bright wire surface before WECMM. (**c**) SEM image of the wire after long-duration WECMM using a traditional unipolar pulse and (**d**) corresponding EDX results for the insoluble products on the surface of the wire.

Comparative machining experiments were performed using a traditional unipolar pulse during WECMM at a feed rate of 0.1 μm/s. Figure 4a shows the result of the short- duration WECMM conducted for approximately 30 min. The width homogeneity of the micro-slit was highly consistent, with an obtained standard deviation of 0.45 μm. Figure 4b shows the result of the long-duration WECMM in a short-circuit position conducted for 6.1 h (approximately 6.0 h). The width homogeneity of the micro-slit was considerably poor, with an obtained standard deviation of 1.21 μm. This is because the bubbles generated during machining gradually accumulated around the machining gap area with continuous machining, and they could not be easily dispersed until they were large enough to automatically rupture. This led to a reduced mass transfer efficiency in the machining gap area that in turn led to easy adsorption of the insoluble electrolytic products on the surface of the wire electrode. Thus, the machining stability and accuracy were decreased. This was confirmed from the real-time CCD images shown in Figure 5, obtained during the long-duration WECMM. This was also confirmed by comparing the clean tungsten wire surface before machining and that with the adsorbed insoluble electrolytic products after short-circuiting. Insoluble electrolytic products are usually composed of metal hydroxide precipitation and insoluble metal particle precipitation, as shown in Figure 6.

According to Bi et al. [2], similar results could be obtained at a feed rate of 0.15 μm/s under the same machining conditions, indicating that the machining accuracy will be reduced in long-duration WECMM of pure aluminum using unipolar pulses compared to that of short-duration WECMM. At the same time, the machining stability was not ideal.

### 4.2. Improving Machining Stability in Long-Duration WECMM Using Bipolar Nanosecond Pulses

**Figure 7:**
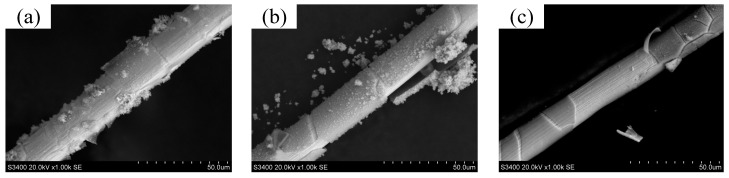
Tungsten wires in the working zone during long-duration WECMM using bipolar nanosecond pulses: (**a**) −0.5 V, (**b**) −1.0 V, and (**c**) −1.5 V.

Figure 7a–c show tungsten wires used in the working zone during long-duration WECMM with bipolar nanosecond pulses. The feed rate was maintained at 0.1 μm/s, and the negative voltages were −0.5, −1.0, and −1.5 V, respectively. The experiments lasted for 8 h without short circuits. Examining the tungsten wires used in the working zone revealed an obvious reduction in the electrolysis products adsorbed on the wire surface. Therefore, using a negative pulse can reduce or inhibit the deposition of electrolysis products on the wire surface, thus improving the machining stability. According to the research of Meng et al. and Gao et al. [15,16], after using bipolar nanosecond pulses for a long-duration wire electrochemical micromachining, the wire in the machining area will be dissolved, resulting in a decrease in the diameter of the wire, which will have adverse effects on the machining. Figure 7b,c show a reduction in the diameter of the wire, attributable to the dissolution of the wire under the action of the negative pulse of −1.0 V and −1.5 V. Therefore, a negative pulse of −0.5 V was considered suitable for the comparative experiments in this study.

**Figure 8:**
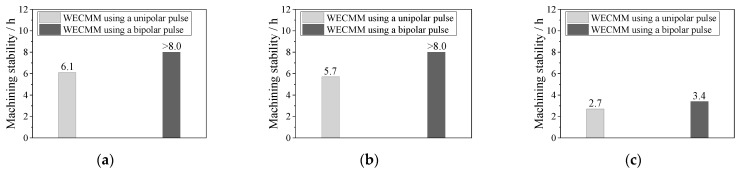
Comparison of machining stability obtained during WECMM using unipolar and bipolar pulses at different feed rates: (**a**) 0.10 μm/s, (**b**) 0.15 μm/s, and (**c**) 0.25 μm/s.

Similar experiments were performed to verify the machining stability in long-duration WECMM using a negative pulse of −0.5 V. The feed rates were maintained at 0.15 and 0.25 μm/s. The machining process lasted for 8 h without short circuits when the feed rate was maintained at 0.15 μm/s, and a short circuit occurred at 3.4 h when the feed rate was maintained at 0.25 μm/s. According to Bi et al. [2], a short circuit occurred after 5.7 h of WECMM using a traditional unipolar pulse when the feed rate was maintained at 0.15 μm/s and after 2.7 h of WECMM using a traditional unipolar pulse when the feed rate was maintained at 0.25 μm/s. Figure 8 compares the machining stabilities achieved using different feed rates. The results show that the machining stability significantly improved in long-duration WECMM using bipolar nanosecond pulses.

### 4.3. Improving Machining Accuracy in Long-Duration WECMM Using Bipolar Nanosecond Pulses

**Figure 9:**
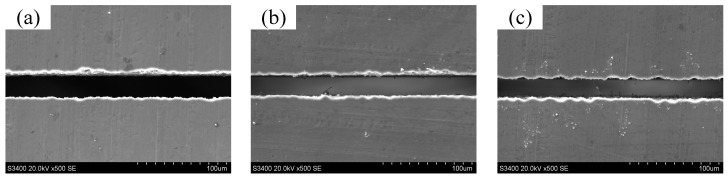
Machined micro-slits in long-duration WECMM using a negative pulse of −0.5 V: machining locations selected for machining durations of approximately (**a**) 5.9, (**b**) 5.7, and (**c**) 2.7 h.

**Figure 10:**
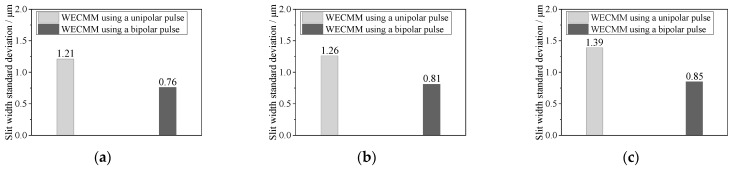
Comparison of machining accuracies obtained during WECMM using unipolar and bipolar pulses at different feed rates: (**a**) 0.10, (**b**) 0.15, and (**c**) 0.25 μm/s.

Figure 9a–c show the machined micro-slits in long-duration WECMM using a negative pulse of −0.5 V. The feed rates were maintained at 0.10, 0.15, and 0.25 μm/s, respectively. According to Bi et al. [2], the standard deviations of the micro-slit widths at the short-circuit locations were 1.26 and 1.39 μm when the feed rates were maintained at 0.15 and 0.25 μm/s, respectively. Therefore, the machining locations were selected for machining durations of 5.9 (approximately 6.0), 5.7, and 2.7 h, respectively. Figure 10 compares the standard deviations of the micro-slit widths. These results show that the machining accuracy significantly improved in long-duration WECMM using bipolar nanosecond pulses.

## 5. Conclusions

The improvements in machining accuracy and stability obtained during long-duration WECMM of pure aluminum using bipolar nanosecond pulses have been experimentally verified. The main conclusions can be summarized as follows:

The obtained width standard deviation of the micro-slit was increased from 0.45 μm to 1.21 μm during WECMM using a traditional unipolar pulse at a feed rate of 0.1 μm/s, indicating that the machining accuracy was reduced in long-duration WECMM using unipolar pulses compared to that in short-duration WECMM.

During long-duration WECMM using a negative pulse of −0.5 V, when the feed rates were maintained at 0.10 and 0.15 μm/s, the machining process without a short circuit exceeded 8 h, and when the feed rate was maintained at 0.25 μm/s, the machining process without a short circuit was increased to 3.4 h.

The comparison of machining accuracies obtained during WECMM using unipolar and bipolar pulses at different feed rates showed that the machining accuracy was significantly improved in long-duration WECMM using bipolar nanosecond pulses.

## Figures and Tables

**Table 1:** Experimental conditions.

Parameter	Value
Wire electrode diameter	20 μm
Workpiece thickness	90 μm
Applied positive voltage	5 V
Applied negative voltage	0 to −1.5 V
Wire vibration amplitude	150 μm
Wire vibration frequency	2 Hz
Feed rate	0.10, 0.15, and 0.25 μm/s
Pulse width and period	50 ns and 5 μs
